# Association Study of Genetic Variants in Autophagy Pathway and Risk of Non-syndromic Cleft Lip With or Without Cleft Palate

**DOI:** 10.3389/fcell.2020.00576

**Published:** 2020-07-14

**Authors:** Shu Lou, Lan Ma, Shiyi Kan, Xin Yu, Yuting Wang, Fan Yang, Guirong Zhu, Liwen Fan, Dandan Li, Hua Wang, Wei Wang, Weibing Zhang, Lin Wang, Yongchu Pan

**Affiliations:** ^1^Jiangsu Key Laboratory of Oral Diseases, Nanjing Medical University, Nanjing, China; ^2^Department of Orthodontics, Affiliated Hospital of Stomatology, Nanjing Medical University, Nanjing, China; ^3^Department of Environmental Genomics, School of Public Health, Nanjing Medical University, Nanjing, China; ^4^State Key Laboratory of Reproductive Medicine, Nanjing Medical University, Nanjing, China

**Keywords:** autophagy, non-syndromic cleft lip with or without cleft palate, single nucleotide polymorphism, molecular genetics, *HIF1A*

## Abstract

Although genetic variants in autophagy pathway genes were associated with the risk of oral cancers and early development in embryos, their associations with non-syndromic cleft lip with or without cleft palate (NSCL/P) risk remained unclear. A two-stage case-control study (2,027 NSCL/P cases and 1,843 controls) was performed to investigate the associations between single nucleotide polymorphisms (SNPs) in 23 autophagy pathway genes and NSCL/P susceptibility. The logistic regression model was used to calculate effects of SNPs on NSCL/P susceptibility. Gene-based analysis was performed via the sequence kernel association test (SKAT) and multi-marker analysis of genomic annotation (MAGMA) methods. Expression quantitative trait loci (eQTL) analysis was conducted using NSCL/P lip tissue samples. Gene expression during embryonic development was evaluated using RNA-Seq. Functional roles were explored by luciferase activity assay, cell apoptosis, proliferation, and cycle *in vitro*. Rs2301104 in *HIF1A* was significantly associated with NSCL/P susceptibility in the combined analysis (OR: 1.29, 95% CI: 1.09–1.29, *P* = 3.39 × 10^–03^), and showed strong evidence of association heterogeneity (*P* = 9.06 × 10^–03^) with obvious association in the female (OR: 1.80; 95% CI: 1.32–2.45; *P* = 1.79 × 10^–04^). The G allele of rs2301104 was associated with enhanced transcription activity and high expression of *HIF1A* compared with that of C allele. Moreover, rs2301104 exhibited an eQTL effect for *HIF1A* with its GC/CC genotypes associated with decreased *HIF1A* expression compared with those with GG genotypes (*P* = 3.1 × 10^–2^). Knockdown of *HIF1A* induced cell apoptosis and inhibited cell proliferation in human embryonic palate mesenchyme (HEPM) and human oral epithelium cells (HOEC). This study demonstrated that rs2301104 in autophagy pathway gene *HIF1A* was associated with susceptibility of NSCL/P.

## Introduction

Non-syndromic cleft lip with or without cleft palate (NSCL/P) is one of the most common human birth defects contributing huge health and financial burdens to the affected individuals, families, and societies (Mangold et al., 2010; Heike and Evans, 2016). It occurs in ~1 in 700 live births worldwide, with prevalence varying by population (Birnbaum et al., 2009).

The occurrence of NSCL/P results from failure of the facial processes to grow or fuse appropriately during early embryologic development (between the 4th and 12th week of gestation) (Beaty et al., 2016; Li et al., 2017). Signaling pathways, including Bmp, Fgf, Shh, and Wnt signaling pathways, are critical for proper lip fusion (Jiang et al., 2006). Similarly, the growth of the palatal shelves is also mediated by epithelial-mesenchymal interactions regulated by multiple signaling pathways and transcriptional factors (Lan et al., 2015). Cell migration, proliferation, and apoptosis in both epithelial cells and cranial neural crest (CNC)-derived mesenchymal cells are involved in mechanisms leading to cleft lip and palate (Tian et al., 2017).

The underlying etiology is complex and multifactorial with a wide range of influences including genetic and environmental factors. Extensive human genetic studies and animal studies have attempted to identify the genetic variants associated with NSCL/P risk (Leslie et al., 2016; Hammond et al., 2018; Reynolds et al., 2019). For instance, Leslie et al. (2016) conducted a multiethnic genome-wide association study in 6480 participants and revealed novel associations on 2p24 near *FAM49A*, and 19q13 near *RHPN2*. Hammond et al. demonstrated that ectopic Hh-Smo signaling downregulates Wnt/BMP pathways, resulting in cleft palate and defective osteogenesis (Hammond et al., 2018).

As a conserved lysosomal degradation process in eukaryotes, autophagy prevents cells from different kinds of stress, such as starvation, hypoxia, or exposure to toxic molecules (Huang and Klionsky, 2007). Defective autophagy is related to physiological and pathological conditions, such as cancer, metabolic, neurodegenerative disease, and aging (Netea-Maier et al., 2016). In the early development stage, autophagy has been shown to be essential in the transition of oocytes to embryos, postpartum survival, development, differentiation and aging in mouse models (Hale et al., 2013). Additionally, autophagy degrades cytoplasmic components, which is vital for embryonic development and human health (Fenouille et al., 2017). Susceptible genes may play essential roles in cell death and inflammation by influencing autophagy in neighboring cells during developmental periods (Lin et al., 2017). Previous studies showed that single nucleotide polymorphisms (SNPs) in autophagy pathway genes were related to the risk of melanoma, neck squamous cell carcinoma, and early embryo development (Capela et al., 2016; White et al., 2016; Fernandez-Mateos et al., 2017), however, their associations with the risk of NSCL/P have never been explored.

Highlighting these bases, with the aim of exploring associations between genetic variants in autophagy pathway and the risk of NSCL/P, we conducted a two-stage case-control study with 2,027 NSCL/P cases and 1,843 controls in the present study. We finally found that rs2301104 in autophagy pathway gene *HIF1A* was associated with susceptibility of NSCL/P. Moreover, we explored the functional roles of the SNP and the gene with *in vivo* and *in vitro* experiments. Our study provides a deeper insight into the roles of autophagy pathway-related genetic variants in the development of NSCL/P.

## Materials and Methods

### Selection of Genes and SNPs From the Autophagy Pathway

The key autophagy associated genes were selected from the Kyoto Encyclopedia of Genes and Genomes (KEGG) and published studies. We obtained 109 genes from the autophagy pathway included in KEGG (map04140), and the following key words were used to search PubMed for publications on or before September 30th, 2018: gene name and craniofacial, craniofacial development, or facial development. Finally, 23 genes related to autophagy and craniofacial development were kept for further analysis (Supplementary Figure 1 and Supplementary Table 1).

**FIGURE 1:**
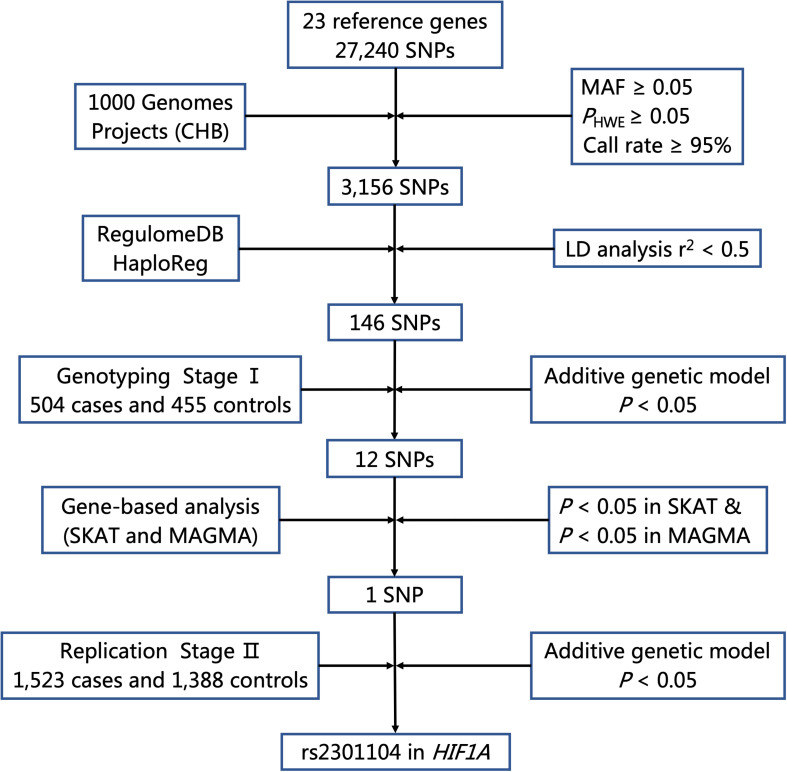
Flow chart for selecting putative functional SNP in autophagy pathway genes. MAF, minor allele frequency; HWE, Hardy–Weinberg Equilibrium; LD, linkage disequilibrium.

The flow chart of selecting putative functional SNPs based on 23 genes we selected is shown in Figure 1. First, a total of 27,240 SNPs in gene regions was selected using the CHB data from the 1000 Genomes Project (Phase I, March 2012) and 3,156 SNPs remained after the following quality control criteria (Wang et al., 2019; Zhu et al., 2019): (a) minor allele frequency (MAF) ≥ 0.05, (b) Hardy-Weinberg equilibrium (HWE) ≥ 0.05, and (c) call rate ≥ 95%. Second, we used RegulomeDB and HaploReg to predict SNP functions. SNPs with Regulome DB Score below five and predicted to hit eQTL effects or bind transcription factors from HaploReg database were retained. Besides, SNPs with pairwise linkage disequilibrium (LD) *r*^2^≥ 0.5 were excluded using PLINK 1.09. Finally, a total of 146 putative functional SNPs in 23 autophagy pathway genes were identified and further explored in a two-stage case-control study.

### Study Participants

We performed a two-stage case-control analysis of NSCL/P in the Chinese Han population. Two stages were totally independent from each other: the stage I was comprised of 504 NSCL/P cases, and 455 newborn controls who were recruited from West China Hospital of Stomatology Sichuan University and the stage II included 1,523 NSCL/P cases and 1,388 controls who were recruited from Affiliated Stomatological Hospital of Nanjing Medical University, Nanjing Children’s Hospital, Xuzhou First People’s Hospital, and Huai’an First People’s Hospital (Supplementary Table 2).

All patients were interviewed and clinically evaluated by an experienced oral surgeon based on detailed diagnostic information from medical records and physical examinations to ensure that individuals with other congenital anomalies, identified CL/P syndromes or developmental delays were excluded from this study. Venous blood samples were collected from all subjects for genetic analysis. The present study was approved by the Ethics Committee of Nanjing Medical University [NJMUERC (2008) No. 20]. At recruitment, informed written consent was obtained from all the participants or their guardians.

### Genotyping

All samples in stage I were genotyped using Affymetrix Axiom CHB1 & CHB2 arrays. Systematic quality control (Wang et al., 2019; Zhu et al., 2019) was performed to filter SNPs, including *P*_HWE_ ≥ 0.05, MAF ≥ 0.05, and call rate ≥ 95%. Genotyping in stage II was performed by TaqMan-MGB assays on an ABI-Prism 7900 instrument (Applied Biosystems, Foster City, CA). The primers of the Taqman probes are listed in Supplementary Table 3.

### Annotations for Candidate SNPs

SNPs were annotated for potential regulatory functions using RegulomeDB^1^ and HaploReg v4.1^2^. We also evaluated the SNPs in the integrative analysis of 127 reference human epigenomes based on NIH Roadmap Epigenomics database^3^.

### Extraction of Mouse Embryo Tissues and RNA-Seq

We purchased six adult male and twelve adult female C57BL/6 mice from Animal Center of Yangzhou University. Two female mice and one male mouse were placed together in the same cage at 8 p.m. and separated at 8 a.m. the next morning. Embryos were counted as E0.5d in the morning that vaginal plug was checked. Lip and palate tissues of E10.5d, E11.5d, E12.5d, E13.5d, E14.5d, and E15.5d embryos were collected. Total RNA was extracted with TRIzol reagent (Invitrogen, Carlsbad, CA, United States) from embryonic tissues of mice, which were approved in quantity and quality by Nanodrop and 1% agarose electrophoresis. mRNA sequencing was performed on Hiseq3000 platform in 10M and 6G depth, respectively. RNA reads were aligned to the mouse genome (MM10) by gSNAP, and the average fragments per kilobase of exon model per million fragments mapped (FPKM) value of all samples was used to normalize mRNA expression.

### *In silico* Gene Expression During Mouse Craniofacial Development and in Human Samples

RNA-Seq data on craniofacial structures during mouse embryo development were downloaded from the FaceBase consortium at FaceBase.org^4^, an online database that describes the overall gene expression during mouse craniofacial development. Count data were normalized using the regularized logarithmic transformation in the DESeq2 R package.

Gene expression in NSCL/P cases and controls of dental pulp stem cells (DPSCs), and mesenchymal stem cells (MSCs) in lip muscle were obtained from Gene Expression Omnibus (GEO) repository (GSE42589 and GSE85748, respectively).

### Cell Culture

The human embryonic palatal mesenchymal (HEPM) cell lines were purchased from American Type Culture Collection (ATCC, Manassas, VA, United States), and cultured in Eagle’s Minimum Essential Medium (ATCC), supplemented with 10% fetal bovine serum (FBS, Gibco), 100 units/ml antibiotics at 37°C under 5% CO_2_. The human oral epithelial cells (HOEC) were obtained from BeNa Culture Collection (Beijing, China), and cultured in Dulbecco’s Modified Eagle Medium (Gibco) supplemented with 10% FBS (Gibco) and 100 units/ml antibiotics and maintained at 37°C under 5% CO_2_.

### Luciferase Activity Assay

In order to evaluate whether there is a difference in the enhancer activity in *HIF1A* of different alleles, we performed the luciferase activity assay. A sequence of 1,000-bp containing rs2301104 G or C allele and *HIF1A* promoter region were synthesized and cloned into the *Nhe*I and *Xho*I restriction sites of the pGL3-basic vector (Promega). HEPM and HOEC cells were seeded at in 24-well culture plates, and each well was transiently transfected with reporter plasmids using Lipofectamine 2000 (Invitrogen, Carlsbad, CA, United States) according to the manufacturer’s instructions. All plasmids were co-transfected with 10 ng pRL-SV40, which contained the *Renilla* luciferase gene. The luciferase activities were measured 48 h after transfection with a dual-luciferase reporter assay system (Promega). The ratio of Firefly luciferase to *Renilla* luciferase activity was assessed. The transfection experiments were performed in triplicate.

### RNA Extraction and Quantitative Real-Time PCR

To compare gene expression of cells and tissues with different genotypes, we constructed the pcDNA3.1-rs2301104 G and pcDNA3.1-rs2301104 C vectors, which were transfected into cells and harvested for 48 h. Total RNA was extracted from cells and 68 NSCL/P lip tissue samples to reverse transcription and PCR reactions using PrimeScript^TM^ RT-PCR kit (TaKaRa, Shiga, Japan). The relative mRNA expression level of *HIF1A* and the internal control *GAPDH* were quantified using ABI 7900 Real-Time PCR system (Applied Biosystems). The primers were listed as follows: *HIF1A* (forward: TCAGGACACAGATTTAGACTTGGAG, reverse: AGTGGTAG TGGTGGCATTAGCA) and *GAPDH* (forward: GGACCTGAC CTGCCGTCTAG, reverse: GTAGCCCAGGATGCCCTTGA). All reactions were conducted in triplicate, and the data were analyzed by the 2^–ΔΔCt^ method. Data are shown as the mean ± standard deviations (SD).

### Small Interference RNA (siRNA) Constructs and Transfection

The small interference RNA (siRNA) oligonucleotides including *HIF1A* siRNA-378 (5′-CCAGAUCUCGGCGAAGUAATT-3′, 5′-UUACUUCGCCGAGAUCUGGTT-3′), *HIF1A* siRNA-2107 (5′-CCAGCAGACUCAAAUACAATT-3′, 5′-UUGUAUUUGAG UCUGCUGGTT-3′), *HIF1A* siRNA-2637 (5′-GCUACUACAUC ACUUUCUUTT-3′, 5′-AAGAAAGUGAUGUAGUAGCTT-3′) and control siRNA (5′-UUCUCCGAACGUGUCACGUTT-3′, 5′-ACGUGACACGUUCGGAGAATT-3′) were designed and purchased from GenePharma (Shanghai, China). Transfection of siRNAs with final concentration 100 nM was performed with Lipofectamine 2000 (Invitrogen, Carlsbad, CA, United States) following the manufacturer’s instructions. Cells were harvested 48 h later for further experiments.

### Cell Apoptosis, Cell Cycle, and Cell Proliferation Assay

Cells were treated with trypsin-EDTA (Gibco) and resuspended as single-cell suspension 48 h after transfection. We stained cells with Annexin V:PE Apoptosis Detection Kit (BD Biosciences, San Jose, CA, United States) for cell apoptosis analysis and analyzed using a Fluorescence Activated Cell Sorting (FACS) System by BD Biosciences (San Jose, CA, United States). As for cell cycle analysis, cells were then fixed in 70% ethanol overnight at 4°C after digested. Propidium iodide (PI)/RNase staining was conducted on fixed cells for flow cytometric analysis. Data were analyzed with FlowJo software (TreeStar, Ashland, OR, United States). Cell proliferation was assessed by absorbance using a Cell Counting Kit-8 assay (CCK8, Dojindo, Kumamoto, Japan) according to the manufacturer’s instructions. Cells were seeded in 96-well plates at a density of 3 × 10^3^ cells per well, approximately. CCK-8 reaction solution was added into each well for 2 h incubation in new medium containing. The absorbance was measured on a spectrophotometer microplate reader (Multiskan MK3, Thermo) at a wavelength of 450 nm. All data are shown as the mean ± SD.

### Statistical Analysis

For evaluating the association between NSCL/P risk and genetic variants, logistic regression analysis under additive model with adjustment for gender was implemented to calculate the crude and adjusted odds ratios (ORs) and their 95% confidence intervals (CIs) (Wang et al., 2016; Zhu et al., 2019). Haplotype analysis were performed using Haploview software with parameters of MAF ≥ 5% and pairwise r^2^ threshold of 0.8 (Barrett et al., 2005). The epistasis test between SNPs in the candidate gene was performed using R software, which was computed by an unconditional logistic regression model specifies the log-odds as: logit(P) = β_0_ + β_G_(G_i_) + β_G×G_(G_i_*G_i_). Goodness-of-fit Chi-square test was performed to test HWE in control groups. We used the sequence kernel association test (SKAT) (Timbers et al., 2016) and Multi-marker Analysis of GenoMic Annotation (MAGMA) tool (de Leeuw et al., 2015) for gene-based analysis. Combined analysis of two stages was performed using a fixed-effect model in R software (Dai et al., 2019). The measure of heterogeneity was tested using Cochran’s Q statistics and *I*^2^. Gene-by-sex (G × S) interaction was computed by an unconditional logistic regression model specifies the log-odds as: logit(P) = β_0_ + β_G_(G_i_) + β_G×E_(G_i_*E_i_). For all graphs, statistical analyses were carried out using two-tailed, unpaired Student’ s *t*-test. Before *t*-test, normal distribution of all the data were checked using normality test and equality of variances were checked using *F*-test. All statistical analyses were performed by R software 3.4.2^5^ and PLINK 1.09. Data were considered statistically significant when *P*-value < 0.05.

## Results

### Association Between Genetic Variants of Autophagy Pathway Genes With the Risk of NSCL/P

As shown in Figure 1, 27,240 SNPs in 23 genes related to autophagy pathway were identified. Among them, 146 putative functional SNPs were picked out, and their associations with risk of NSCL/P were examined in the stage I that was comprised of 504 NSCL/P cases and 455 newborn controls. Twelve SNPs (rs77141447, rs16822638, rs7625881, rs14016, rs12813551, rs147920828, rs2301104, rs4776786, rs80225705, rs3784605, rs7182342, and rs2283791) were significantly associated with the risk of NSCL/P in additive logistic model with the adjustment of gender (Table 1).

**TABLE 1:** The association of 12 significant SNPs with the risk of NSCL/P.

**Chr**	**SNP**	**Gene**	**Allele^a^**	**Call rate (%)**	**MAF**		**Genotype distribution^b^**		***P*_hwe_**	**OR (95% CI)**	***P***	**OR (95% CI)^c^**	***P*^c^**
											
					**Case**	**Control**	**Case**	**Control**					
2	rs77141447	*IRS1*	C/A	96.976	0.071	0.049	421/68/1	397/43/0	0.25	1.53 (1.02–2.28)	3.84E−02	1.50 (1.00–2.24)	4.91E−02
2	rs16822638	*IRS1*	A/G	100.000	0.217	0.177	303/183/18	313/123/19	1.00	1.29 (1.03–1.63)	2.70E−02	1.30 (1.04–1.64)	2.32E−02
3	rs7625881	*ATG7*	A/G	95.620	0.373	0.318	184/235/62	199/197/40	0.24	1.29 (1.06–1.58)	1.11E−02	1.29 (1.05–1.57)	1.42E−02
3	rs14016	*ATG7*	C/T	100.000	0.432	0.380	161/251/92	168/228/59	0.32	1.25 (1.04–1.50)	2.03E−02	1.25 (1.03–1.50)	2.17E−02
12	rs12813551	*KRAS*	T/C	99.896	0.194	0.244	329/153/21	270/148/37	0.57	0.76 (0.62–0.94)	1.08E−02	0.75 (0.61–0.93)	7.95E−03
12	rs147920828	*KRAS*	C/T	96.767	0.093	0.131	403/83/4	333/95/10	0.40	0.68 (0.51–0.91)	9.94E−03	0.67 (0.50–0.90)	7.67E−03
14	rs2301104	*HIF1A*	G/C	100.000	0.100	0.073	408/91/5	393/58/4	0.42	1.41 (1.03–1.95)	3.48E−02	1.39 (1.01–1.92)	4.55E−02
15	rs4776786	*MAP2K1*	T/C	98.749	0.073	0.050	427/66/3	409/39/3	0.16	1.47 (1.01–2.14)	4.64E−02	1.48 (1.02–2.17)	4.15E−02
15	rs80225705	*IGF1R*	C/T	96.976	0.090	0.065	418/81/5	396/59/0	1.00	1.44 (1.02–2.03)	3.80E−02	1.44 (1.02–2.04)	3.71E−02
15	rs3784605	*IGF1R*	T/C	100.000	0.153	0.115	370/114/20	354/97/4	0.07	1.36 (1.05–1.76)	2.06E−02	1.34 (1.04–1.75)	2.66E−02
15	rs7182342	*IGF1R*	C/G	96.976	0.314	0.367	236/220/48	187/202/66	0.57	0.79 (0.65–0.95)	1.44E−02	0.79 (0.65–0.96)	1.54E−02
22	rs2283791	*MAPK1*	C/G	99.270	0.165	0.204	342/151/7	286/148/18	0.16	0.76 (0.60–0.97)	2.64E−02	0.77 (0.61–0.99)	3.73E−02

*NSCL/P, non-syndromic cleft lip with or without cleft palate; Chr, chromosome; SNP, single nucleotide polymorphism; MAF, minor allele frequency; OR, odds ratio; CI, confidence interval. ^*a*^Alleles: shown as major/minor allele. ^*b*^Major homozygote/heterozygote/minor homozygote. ^*c*^OR (95% CI) and P values were derived from logistic regression analysis with adjustment for gender under the assumption of an additive genetic model.*

### Gene-Based Analysis With the Risk of NSCL/P

To evaluate the joint effects of common SNPs in a gene unit, we further performed gene-based analysis using SKAT and MAGMA methods to identify potentially associated genes and found that *HIF1A* (*P*_SKAT_ = 9.42 × 10^–03^, *P*_MAGMA_ = 2.74 × 10^–02^), *BCL2L1* (*P*_SKAT_ = 1.05 × 10^–02^) and *KRAS* (*P*_MAGMA_ = 1.76 × 10^–02^) were significantly associated with NSCL/P susceptibility. Only *HIF1A* achieved consistent associations in both methods (Supplementary Table 4); thus, rs2301104 in *HIF1A* remained for further analysis.

### Replication and Combined Analysis of rs2301104 With the Risk of NSCL/P

Rs2301104 was genotyped for validation with an additional 1,523 NSCL/P case and 1f88 control subjects and showed significant association with the same direction of effect as that observed in stage I (OR: 1.39; 95% CI: 1.01–1.92; *P* = 4.55 × 10^–02^ in stage I, OR: 1.25; 95% CI: 1.03–1.53; *P* = 2.72 × 10^–02^ in stage II, Table 2). Similar results were also observed in the combined analysis from the two stages (OR: 1.29; 95% CI: 1.09–1.53; *P* = 3.39 × 10^–03^, Table 2). No significant heterogeneity for rs2301104 was observed among different stages (*P*_het_ = 0.70, Table 2). Further, to explore whether rs2301104 participated in development of NSCL/P alone or not, we also performed haplotype and epistasis analysis. No significant haplotype and SNPs interacted with rs2301104 was found, which indicated rs2301104 may play an independent role in development of NSCL/P (Supplementary Figure 2 and Supplementary Table 5).

**TABLE 2:** Association between rs2301104 in *HIF1A* and the risk of NSCL/P.

**Loci**	**SNP**	**Position (hg19)**	**Gene**	**Alleles^a^**	**Stage**	**Call rate (%)**	**MAF (Case/Control)**	**GG/GC/CC**		**OR (95% CI)^b^**	***P*^b^**	***P*_het_^c^**
								**Case**	**Control**			
14q23.2	rs2301104	62165028	*HIF1A*	G/C	Stage I	100.000	0.100/0.073	408/91/5	393/58/4	1.39 (1.01–1.92)	4.55E−02	–
					Stage II	98.111	0.075/0.060	1256/201/11	1240/129/19	1.25 (1.03–1.53)	2.72E−02	–
					Combine	–	–	–	–	1.29 (1.09–1.29)	3.39E−03	0.70

*NSCL/P, non-syndromic cleft lip with or without cleft palate; SNP, single-nucleotide polymorphism; MAF, minor allele frequency; OR, odds ratio; CI, confidence interval. ^*a*^Alleles: shown as major/minor allele. ^*b*^OR (95% CI) and P values were derived from logistic regression analysis with adjustment for gender under the assumption of an additive genetic model. ^*c*^P-value of heterogeneity test.*

### Gender Stratification Analysis of rs2301104 With the Risk of NSCL/P

**FIGURE 2:**
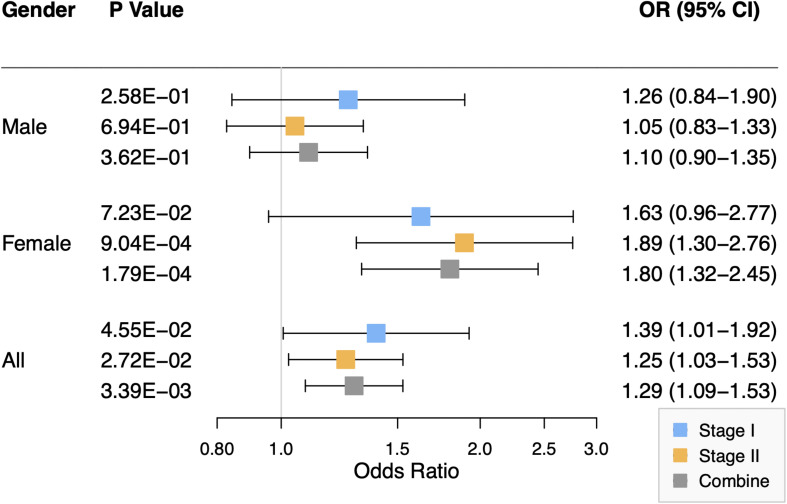
Forest plot of the association between rs2301104 with risk of NSCL/P in stage I, stage II, and combined analysis, stratified according to the gender of study participants.

As shown in Figure 2, the association of rs2301104 with the risk of NSCL/P showed strong evidence of heterogeneity (*P*_het_ = 9.06 × 10^–03^) among males and females. Significant association between rs2301104 and NSCL/P risk were observed in the female (OR: 1.80; 95% CI: 1.32–2.45; *P* = 1.79 × 10^–04^), rather than in the male (OR: 1.10; 95% CI: 0.90–1.35; *P* = 0.362, Figure 2). Further, rs2301104 had a significant interaction with gender (*P* = 1.13 × 10^–03^), constituting highly statistically significant evidence of the involvement of rs2301104 with gender preference in NSCL/P.

### Potential Regulatory Role of rs2301104 on HIF1A

To investigate the underlying mechanisms of rs2301104, we conducted functional annotation analysis on it. It was found that rs2301104 resided within chromatin regions expressing marks indicative of enhancer activity, promoter activity, transcription regulatory by predictions from Haploreg, RegulomeDB, UCSC Genome Browser, and Roadmap Epigenomics Project (Supplementary Table 6 and Supplementary Figure 3).

**FIGURE 3:**
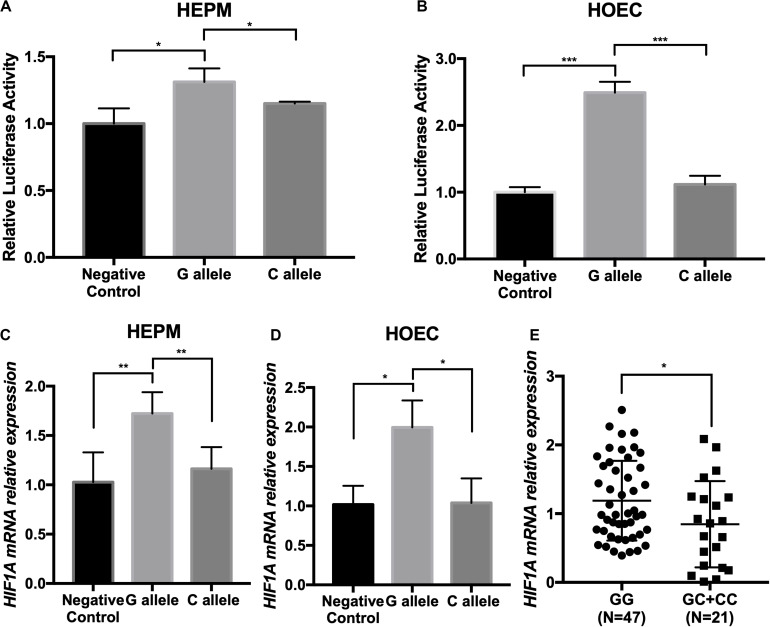
The rs2301104 alleles affect the enhancer activity and expression of *HIF1A*. **(A)** HEPM and **(B)** HOEC cells were transiently transfected with rs2301104 [G], rs2301104 [C], and *HIF1A* promoter with PGL3-basic luciferase reporter vector. pRL-SV40 was co-transfected into cells. The activity of luciferase was assayed 48h later. qRT-PCR analysis of *HIF1A* expression at 48 h after plasmids transfection in **(C)** HEPM and **(D)** HOEC cells. It was indicated that the expression of *HIF1A* was lower with C allele constructs, compared with G allele constructs. Results are shown as mean values with the standard deviation (SD) normalized to *GAPDH.* **(E)** *HIF1A* mRNA expression was detected by qRT-PCR in 68 NSCL/P lip tissue samples. The *HIF1A* expression was significantly lower in samples with GC/CC genotypes than those with GG genotypes. The results were normalized to *GAPDH*. The *P*-value was calculated with two-side *t*-test (*n* = 6; *P < 0.05, **P < 0.01, ***P < 0.001).

We thereby constructed enhancer luciferase reporter vectors containing the rs2301104-centered region and the *HIF1A* promoter and then tested the luciferase activity 48 h after transfecting different plasmids in HEPM and HOEC cells. The rs2301104 G allele revealed a significantly increased enhancer activity compared with that of C allele (Figures 3A,B). This result was also supported in HEPM and HOEC cells that the mRNA expression of *HIF1A* was lower with C allele constructs, compared with G allele constructs (Figures 3C,D).

To evaluate the effect on rs2301104 in *HIF1A*, we further performed an eQTL analysis in lip tissue samples from 68 NSCL/P patients and found that rs2301104 was significantly associated with the expression of *HIF1A* (*P* = 3.1 × 10^–2^). Expression of *HIF1A* was higher in samples of GG genotypes compared with those of GC/CC genotypes (Figure 3E).

### *HIF1A* Expression During Mouse Craniofacial Development and in Human Samples

We detected continuous expression of *HIF1A* in the mouse lip and palate development during E10.5d to E15.5d as well as craniofacial structures from E10.5d to E14.5d (Supplementary Figure 4), implicating its essential role in the craniofacial development. In addition, we found that the expression of *HIF1A* was decreased in the DPSCs, and MSCs in lip muscle of NSCL/P cases, compared with those in controls (*P* = 8.37 × 10^–2^ and *P* = 3.74 × 10^–1^, respectively, Supplementary Figure 5).

### Functional Analyses of *HIF1A in vitro*

**FIGURE 4:**
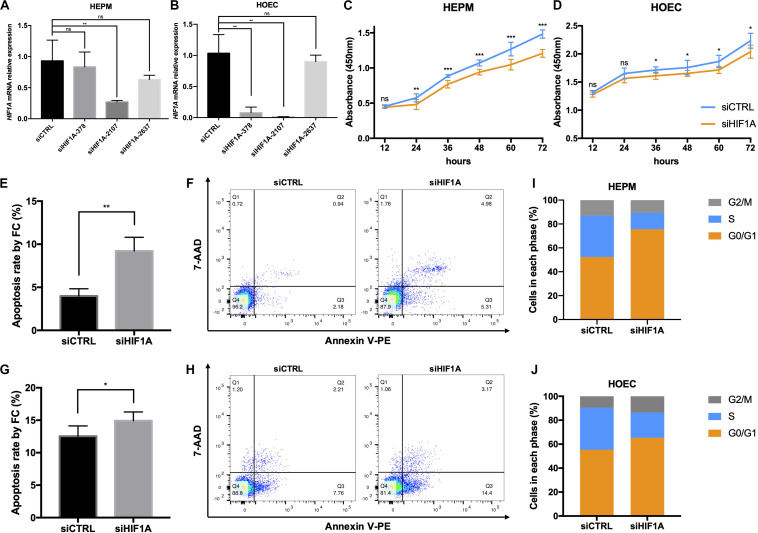
*In vitro* functional roles of *HIF1A* in HEPM and HOEC. **(A,B)** qRT-PCR analysis of the *HIF1A* knockdown efficiency in HEPM and HOEC cells, respectively (*n* = 6). **(C,D)** Cell counting kit-8 assay was used to assess the proliferation of HEPM and HOEC cells after transfection with *HIF1A* siRNAs (*n* = 6). **(E–H)** Quantitative analysis of cell apoptosis by flow cytometry between the two groups (*n* = 6). The rate of apoptosis is higher in **(E,F)** HEPM cells, and **(G,H)** HOEC cells with *HIF1A* knockdown than in the corresponding negative controls. **(I,J)** Effects of *HIF1A* knockdown on the cell cycle in HEPM and HOEC cells (*n* = 6). (ns, no significance, *P < 0.05, **P < 0.01, ***P < 0.001).

To further address the function of *HIF1A*, we knocked down the expression of *HIF1A* in HEPM and HOEC cells through the transfection of *HIF1A* siRNA-2107 given its highest efficiency (Figures 4A,B). Flow cytometric analysis revealed a significantly increased apoptosis rate in cells transfected with *HIF1A* siRNA-2107 (Figures 4E–H). The results of CCK-8 assays showed that knockdown of *HIF1A* significantly inhibited cell proliferation (Figures 4C,D). Moreover, the cell cycle assay showed that knockdown of *HIF1A* increased the number of G0/G1 phase cells, with a significant reduction in the number of S phase cells (Figures 4I,J).

## Discussion

Autophagy participates in many pathological conditions, such as neurodegeneration, autoimmune disease, cancer, and Crohn’s disease (Nyfeler and Eng, 2016). In the present study, we evaluated the relationships between SNPs on core genes of autophagy pathway with risk of NSCL/P by a two-stage case-control study. Using association and gene-based analysis, we found that rs2301104 in *HIF1A* was significantly associated with NSCL/P risk. Notably, gender stratification analysis indicated that rs2301104 exhibited a significant association in the females but not males. Given several possible mechanisms, the underlying biochemical causes might involve sex hormones, sex-biased methylation (McCarthy et al., 2014). Moreover, autophagy is differentially regulated by sex, and the interaction between sex, sex-hormones, and tissue variability in regulating the heat shock response pathway was also observed (Tower et al., 2020).

Rs2301104 located in the first intron of *HIF1A* with enriched enhancer histone marks (including H3K4me1, H3K4me3, and H3K27ac) and previous studies had proven that SNPs in this region played essential roles in gene transcription and expression. For instance, Wang et al. identified a variant at 12p13.2 with enriched histone marks associated with colorectal cancer risk by affecting the binding affinity of transcriptional factor (Wang et al., 2016). Here, luciferase activity assay showed that rs2301104 C allele significantly decreased transcription activity, compared with that of G allele. Further, the rs2301104 C allele was associated with lower expression level of *HIF1A* mRNA in HEPM and HOEC cells. These findings suggested that rs2301104 was involved in transcriptional regulation of *HIF1A*.

We observed expression of *HIF1A* during mouse craniofacial development from E10.5d to E14.5d, implicating its involvement in the development of lip and palate. Notably, individuals carrying rs2301104 GC/CC genotypes (risk genotype) showed significantly lower expression of *HIF1A* than GG genotypes in lip tissue samples and a similar trend was also observed in DPSCs, and MSCs in lip muscle of NSCL/P cases and controls, highlighting the association between low expression of *HIF1A* and increased susceptibility of NSCL/P. We further knockdown of *HIF1A* to evaluate its role in the function of HOEC and HEPM. Promoted cell apoptosis, inhibited cell proliferation, and increase in G0/G1 phase cells, accompanied by a decrease in the number of S phase cells were detected and all of events commonly occurred during the development of NSCL/P (Tian et al., 2017; Lukacs et al., 2019). For instance, Lu et al. reported that cell proliferation increased and apoptosis decreased in the E13d mutant rabbit model related to NSCL/P, which would lead to the persistence of seams in the facial processes (Lu et al., 2019).

*HIF1A* played an important role in terms of embryo development. Severe congenital malformations including heart defects, retinopathy, brain lesions could be detected by culturing embryos at an inappropriate oxygen concentration level, which is mainly regulated by hypoxia-inducible factors (HIFs) (Bohuslavova et al., 2013; Feliciano et al., 2013; Nunes et al., 2015). *Hif1a*-deficient mice exhibited abnormal development of heart morphology and embryonic lethality between E11.0d and E12.0d (Krishnan et al., 2008; Forristal et al., 2010). According to the DECIPHER database, deletion of *HIF1A* resulted in congenital developmental defects, including microcephaly, aplasia of the tongue, and facial asymmetry (Firth et al., 2009).

The exact reason that *HIF1A* contributed to these developmental anomalies remained obscure. However, autophagy, induced by hypoxia whose activation was dependent on *HIF1A* (Liu et al., 2017), may be a possible way. Lu et al. demonstrated that the inhibition of autophagy might cause delayed development by affecting the epithelial-mesenchymal transitions process during chick development (Lu et al., 2014). Previous study showed that the loss of autophagy function in both osteoblasts and osteocytes might lead to a decrease in craniofacial bone mass in mouse models (Thomas et al., 2019). It had been reported that impaired autophagy could cause the loss of the protein quality control mechanism that is at the basis of inherited cardiomyopathies (Dorsch et al., 2019). Based on these studies, we hypothesized that decreased expression of *HIF1A* might break the oxygen homeostatic response during embryonic development, thereby inhibit autophagy and further exacerbate susceptibility to various developmental anomalies, including NSCL/P. A schematic model is shown in Supplementary Figure 6.

Taken together, we identified rs2301104 in autophagy pathway genes *HIF1A* that contributed to the risk of NSCL/P, providing further knowledge about the etiology of the disease. Further studies were warranted to explore the underlying mechanisms.

## Data Availability Statement

All datasets generated for this study are included in the article/Supplementary Material.

## Ethics Statement

The studies involving human participants were reviewed and approved by the Ethics Committee of Nanjing Medical University. Written informed consent to participate in this study was provided by the participants’ legal guardian/next of kin. The animal study was reviewed and approved by the Ethics Committee of Nanjing Medical University.

## Author Contributions

SL analyzed the data, drafted the manuscript, and performed *in vivo* and *in vitro* experiments with SK. LM, WZ, YP, and LW designed and directed the study, obtained the financial support, and critically revised the manuscript. XY performed the genotyping in stage II. YW, LF, and DL were responsible for sample processing. FY and GZ performed the statistical analysis. HW and WW were responsible for subject recruitment and sample collection. All authors read and approved the final manuscript.

## Conflict of Interest

The authors declare that the research was conducted in the absence of any commercial or financial relationships that could be construed as a potential conflict of interest.

## Footnotes

1 http://www.regulomedb.org

2 http://www.broadinstitute.org/mammals/haploreg/haploreg.php

3 http://www.roadmapepigenomics.org/

4 https://www.facebase.org/, GSE67985.

5 http://www.r-project.org/
