# Supplementary material for: Association Study of Genetic Variants in Autophagy Pathway and Risk of Non-syndromic Cleft Lip With or Without Cleft Palate
Source: Front Cell Dev Biol. 2020 Jul 14;8:576. doi: 10.3389/fcell.2020.00576 (PMC7381156; doi:10.3389/fcell.2020.00576)
Supplement: Supplementary file 1 [file Data_Sheet_1.pdf]

Supplementary Table 1 Selected genes in the autophagy pathway

Chr	Gene	Position start (2kb upstream)[a]	Position end[a]	PMID
1	*MTOR*	11164587	11322614	28686577, 29975682
1	*NRAS*	115245084	115259515	23791108, 21263000
2	*IRS1*	227594032	227664545	25308507
3	*ATG7*	11312009	11599139	26243186
3	*RAF1*	12623099	12705700	28548091, 27484170, 29271604
6	*MAP3K7*	91221291	91297020	23546880
6	*ATG5*	106630350	106773695	26243186
7	*RHEB*	151161097	151217010	28457749
9	*TSC1*	135764734	135820094	25639352, 26052552
10	*JNK*	49512681	49647403	25922332, 25008326
10	*PTEN*	89621194	89728532	26229595, 29115005, 28774669
11	*HRAS*	530241	535561	25677562, 24668879, 17250658
11	*TRAF6*	36503316	36531863	29484780, 14699584, 12711536
12	*KRAS*	25356179	25403870	22946697, 22302539, 20944652
13	*LAMP1*	113949435	113977746	20967466, 29683502, 19097165
14	*HIF1A*	62160117	62214977	29791568
14	*EIF2S1*	67825033	67853233	26414443
14	*AKT*	105233685	105262080	29190819, 29115005
15	*MAP2K1*	66677181	66783882	29590634, 18042262
15	*IGF1R*	99190271	99507759	24793337, 21204214
19	*MAP2K2*	4088318	4124126	29590634, 20301365, 29799162
20	*BCL2L1*	30250260	30311752	26575826, 30232009
22	*ERK*	22111945	22221970	29590634, 28548091, 26589919

[a]Based on NCBI build 37 of the human genome.

Supplementary Table 2 Basic characteristics of study subjects

Variables	Stage I		Stage II	
	Cases (N=504)	Controls (N=455[a])	Cases (N=1,523)	Controls (N=1,388)
Age (Mean±SD)	1.51±0.51	0.00±0.00	4.57±6.81	10.80±2.20
Gender, %				
Male	308 (61.1)	236 (51.9)	934 (61.3)	854 (61.5)
Female	196 (38.9)	219 (48.1)	589 (38.7)	534 (38.5)

SD: standard deviation;

[a] newborn infant.

Supplementary Table 3 Sequence of the TaqMan probes and primer for rs2301104

SNP	Primer (5'-3')	Probe (5'-3'-MGB)
rs2301104	F: TTTGGCCATTAAAAGTCAAGAAGCT	G: FAM-TTAAGAGCATAGGATCTAG-MGB
	R: TGGGACCCTTAACTCAGAGGTATC	C: HEX-TTAAGAGCATACGATCTAG-MGB

F: forward primer; R: reverse primer.

Supplementary Table 4 Results of interaction effects with rs2301104 and SNPs in *HIF1A*

Variants	beta†	Standard error†	*P* value†
rs2301104*rs2301105	-0.08511631	0.13969438	0.54232313
rs2301104*rs7143164	-0.07612789	0.12910953	0.55543336
rs2301104*rs74646372	-0.10242131	0.17820329	0.56546430
rs2301104*rs1951795	0.04762555	0.11913767	0.68933900
rs2301104*rs2301107	-0.07467760	0.13937120	0.59208432
rs2301104*rs72714566	0.10150233	0.14127744	0.47247310
rs2301104*rs75332990	-0.05422285	0.13797108	0.69431832
rs2301104*rs12882000	-0.02436723	0.12796579	0.84898015
rs2301104*rs12881961	-0.02213248	0.12819007	0.86292368
rs2301104*rs12232182	0.02750728	0.11761212	0.81507713
rs2301104*rs12435848	0.02750728	0.11761212	0.81507713
rs2301104*rs10459450	-0.01931183	0.12771229	0.87980728
rs2301104*rs10129270	-0.00746117	0.13617557	0.95630515
rs2301104*rs3783752	-0.02061687	0.13222221	0.87609140
rs2301104*rs12887165	-0.04086926	0.13396984	0.76031804
rs2301104*rs72714570	0.07830728	0.14117908	0.57912293
rs2301104*rs1957757	-0.01304121	0.13195044	0.92126993
rs2301104*rs12434438	0.08492532	0.12070465	0.48169470
rs2301104*rs2301108	-0.00479409	0.13203324	0.97103539
rs2301104*rs2301109	-0.01304121	0.13195044	0.92126993
rs2301104*rs11622129	0.05842446	0.12168156	0.63112624
rs2301104*rs11158358	0.04132028	0.12095064	0.73262987
rs2301104*rs2301110	0.05842446	0.12168156	0.63112624
rs2301104*rs2301111	0.08492532	0.12070465	0.48169470
rs2301104*rs966824	-0.06505146	0.13344736	0.62592599
rs2301104*rs75606298	0.08465367	0.12070699	0.48310622
rs2301104*rs7161527	0.05827866	0.12168894	0.63199910
rs2301104*rs2301113	0.08530791	0.10404688	0.41227383
rs2301104*rs4902080	-0.07712862	0.13780135	0.57567813

rs2301104*rs4902081	0.10043617	0.10475481	0.33767271
rs2301104*rs35244739	0.11182181	0.10462780	0.28517865
rs2301104*rs10147275	0.03383609	0.13057690	0.79553670
rs2301104*rs2057482	0.02805423	0.13051822	0.82981024

†beta, standard error, *P* value were estimated using logistic regression model adjusted for gender.

Supplementary Table 5 Gene-based analysis results by SKAT and MAGMA

Gene	Chr	Start site[a]	Stop site[a]	P_{SKAT}[b]	P_{MAGMA}[c]
MTOR	1	11166588	11322614	5.03E-01	3.30E-01
NRAS	1	115247085	115259515	-	-
IRS1	2	227596033	227664545	6.18E-02	3.14E-01
ATG7	3	11314010	11599139	6.83E-01	4.04E-01
RAF1	3	12625100	12705700	8.07E-01	2.02E-01
MAP3K7	6	91223292	91297020	5.16E-02	1.96E-01
ATG5	6	106632351	106773695	7.07E-01	3.80E-01
RHEB	7	151163098	151217010	5.80E-01	7.69E-01
TSC1	9	135766735	135820094	1.10E-01	2.08E-01
JNK	10	49514682	49647403	7.45E-01	7.63E-01
PTEN	10	89623195	89728532	1.34E-01	3.30E-01
HRAS	11	532242	535561	8.45E-01	6.91E-01
TRAF6	11	36505317	36531863	8.38E-01	7.60E-01
KRAS	12	25358180	25403870	8.39E-02	**1.76E-02**
LAMP1	13	113951436	113977746	7.41E-01	4.06E-01
HIF1A	14	62162118	62214977	**9.42E-03**	**2.74E-02**
EIF2S1	14	67827034	67853233	5.32E-01	1.29E-01
AKT	14	105235686	105262080	2.80E-01	4.96E-01
MAP2K1	15	66679182	66783882	1.32E-01	1.85E-01
IGF1R	15	99192272	99507759	6.65E-01	8.20E-01
MAP2K2	19	4090319	4124126	3.87E-01	8.76E-01
BCL2L1	20	30252261	30311752	**1.05E-02**	8.64E-02
ERK	22	22113946	22221970	2.87E-01	3.61E-01

[a]Based on NCBI build 37 of the human genome.

[b]*P* adjusted with gender using SKAT;

[c]*P* adjusted with gender using MAGMA;

Bold values represent significantly difference.

Supplementary Table 6 Function annotation for rs2301104

SNP	Chr	BP[a]	Gene	Allele[b]	Promoter histone marks	Enhancer histone marks	DNAse	Proteins bound	Selected eQTL hits	Motifs changed	Regulome DB Score[c]
rs2301104	14	62165028	*HIF1A* (intron)	G/C	22 tissues	6 tissues	27 tissues	GATA1			4

[a]Based on NCBI build 37 of the human genome;

[b]Major/minor allele;

[c]Regulome DB Score: 4, TF binding + DNase peak.

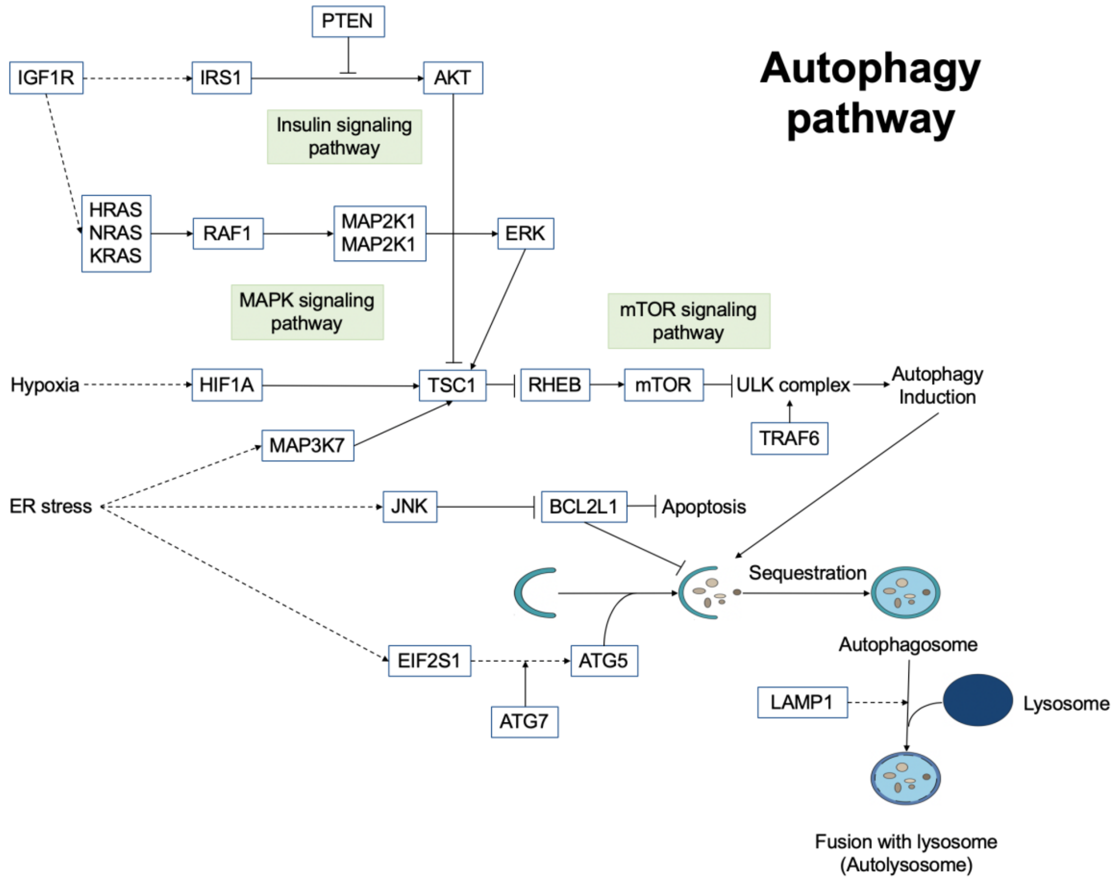

Supplementary Figure 1. Key genes of the autophagy pathway. The boxes with blue solid border show the 23 genes analyzed in this study, and the boxes with green background represent the other signaling pathways involved in the autophagy pathway.

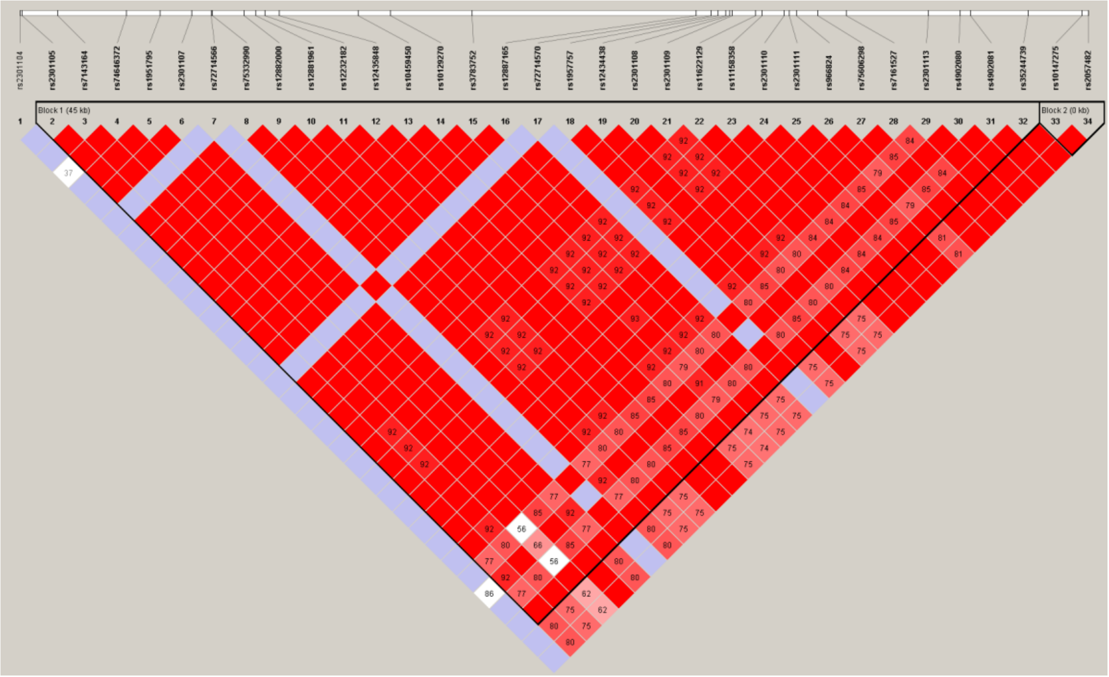

Supplementary Figure 2. Calculated haplotypes within the *HIF1A* gene. Two blocks within the *HIF1A* gene were built using Haploview software. No significant haplotype contains rs2301104 was found, which indicated rs2301104 may play an independent role in development of NSCL/P.

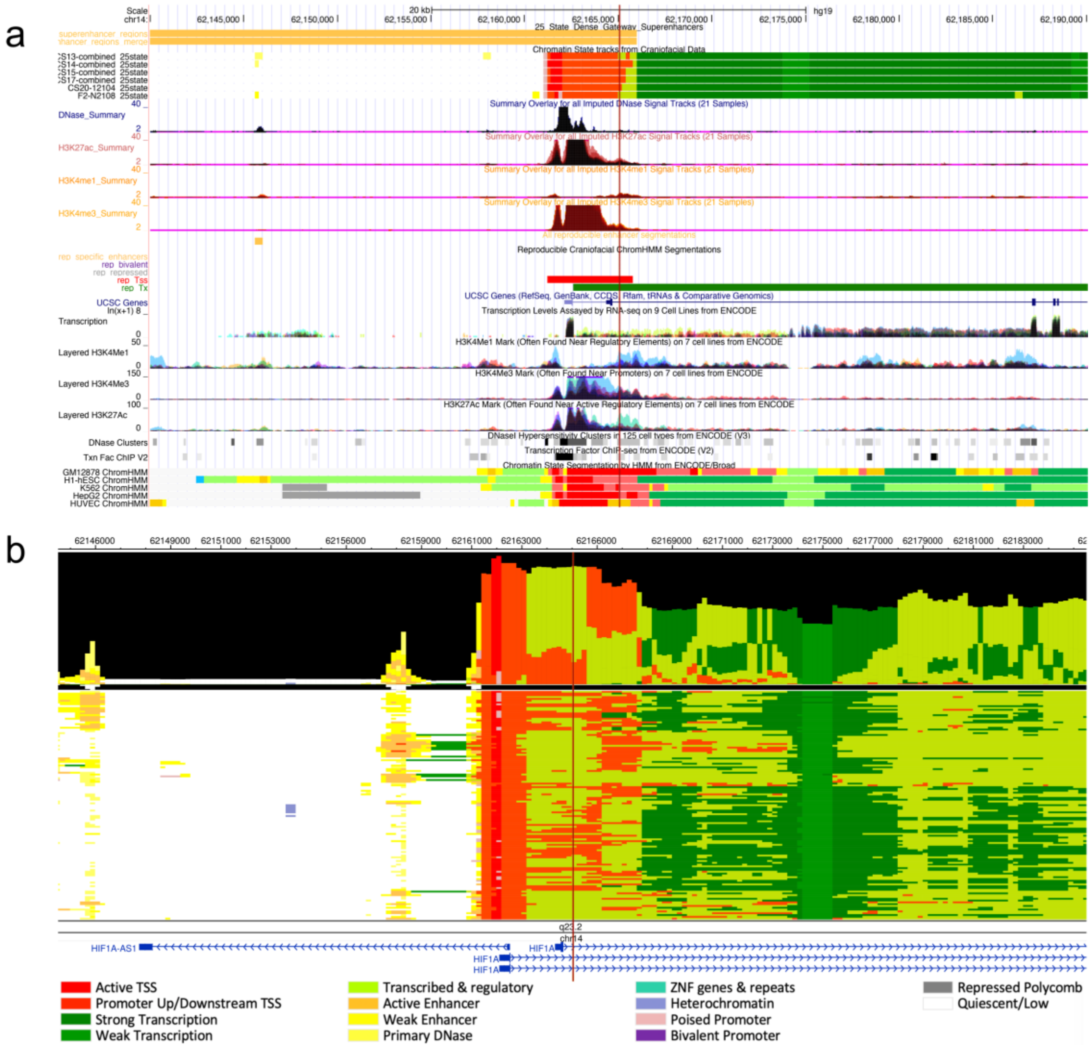

Supplementary Figure 3. Functional annotation of rs2301104. The red vertical line marks rs2301104. Results indicated rs2301104 resided within chromatin regions expressing marks indicative of enhancer activity, promoter activity, transcription regulatory. (a) Enrichment of orofacial clefting and craniofacial measure associations in craniofacial enhancers annotations for early development enhancer state prediction (Upper) and chromatin state segmentation, multiple trackers for H3K4me1, H3K4me3, H3K27Ac histone modifications from ENCODE data provided by the UCSC Genome Browser (Lower) (http://genome.ucsc.edu/index.html). (b) Summary of 127 epigenomes annotations in rs2301104, where vertical stack view of epilogos was generated from a chromatin state model based on imputed data provided by NIH Roadmap Epigenomics Consortium (http://egg2.wustl.edu/roadmap/web_portal). The color legend is at the bottom.

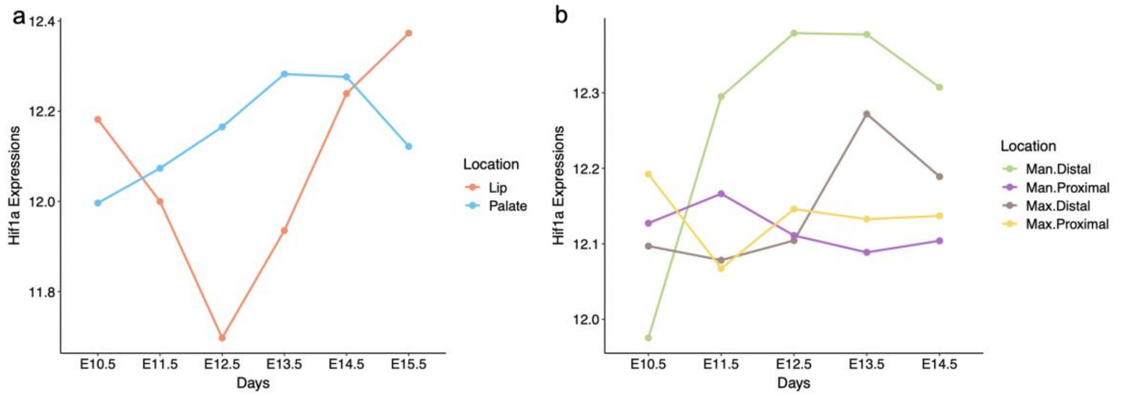

Supplementary Figure 4. (a) Expression levels of Hif1a in mouse lip and palate tissues during mouse embryo development stage from E10.5d to E15.5d by RNA-seq. (b) Expression levels of Hif1a in mouse craniofacial tissues during mouse embryo development stage from E10.5d to E14.5d according to Facebase database (http://www.facebase.org/, GSE67985). Man.Distal: Mandibular distal location; Man.Proximal: Mandibular proximal location; Max.Distal: Maxillary distal location; Max. Proximal: Maxillary proximal location.

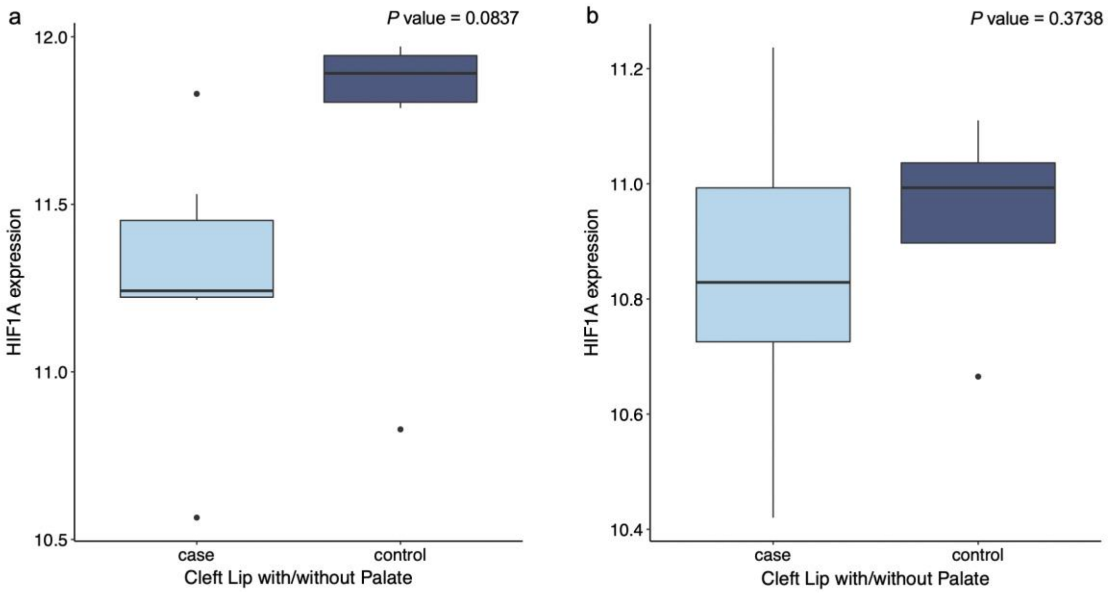

Supplementary Figure 5. Expression levels of *HIF1A* was downregulated in (a) dental pulp stem cells, and (b) mesenchymal stem cells in lip muscle of NSCL/P patients, compared with those in controls. *P* value was determined by an unpaired, two-tailed Student's t test.

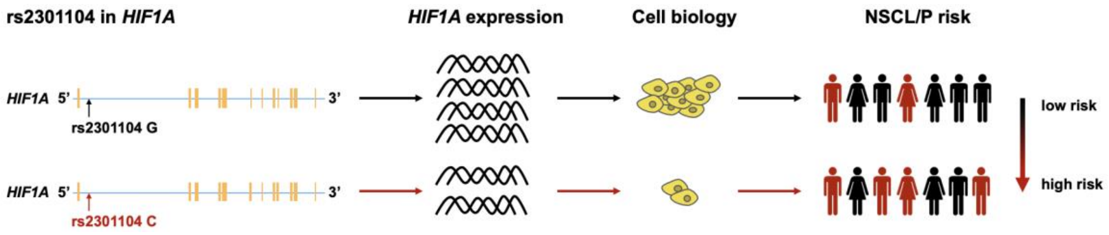

Supplementary Figure 6. A schematic model of our findings. The SNP rs2301104 C allele is associated with an increased risk of NSCL/P by downregulating *HIF1A* expression.
